# Serum Levels of Beta2-Microglobulin and Free Light Chains of Immunoglobulins Are Associated with Systemic Disease Activity in Primary Sjögren’s Syndrome. Data at Enrollment in the Prospective ASSESS Cohort

**DOI:** 10.1371/journal.pone.0059868

**Published:** 2013-05-24

**Authors:** Jacques-Eric Gottenberg, Raphaèle Seror, Corinne Miceli-Richard, Joelle Benessiano, Valerie Devauchelle-Pensec, Philippe Dieude, Jean-Jacques Dubost, Anne-Laure Fauchais, Vincent Goeb, Eric Hachulla, Pierre Yves Hatron, Claire Larroche, Véronique Le Guern, Jacques Morel, Aleth Perdriger, Xavier Puéchal, Stephanie Rist, Alain Saraux, Damien Sene, Jean Sibilia, Olivier Vittecoq, Gaétane Nocturne, Philippe Ravaud, Xavier Mariette

**Affiliations:** 1 Rheumatology Centre National de Référence des Maladies Auto-Immunes Rares, Institut National de la Santé et de la Recherche Médicale UMRS_1109, Fédération de Médecine Translationnelle de Strasbourg, Strasbourg University Hospital, Université de Strasbourg, Strasbourg, France; 2 Rheumatology, Bicetre Hospital, Institut National de la Santé et de la Recherche Médicale U-1012, Université Paris Sud, Assistance Publique des Hôpitaux de Paris, Paris, France; 3 Centre de Ressources Biologiques, Bichat Hospital, Assistance Publique des Hôpitaux de Paris, Paris, France; 4 Rheumatology, Brest University Hospital, Brest, France; 5 Rheumatology, Bichat Hospital, Assistance Publique des Hôpitaux de Paris, Paris, France; 6 Rheumatology, Clermont-Ferrand Hospital, Clermont-Ferrand, France; 7 Internal Medicine, Limoges Hospital, Limoges, France; 8 Rheumatology, Amiens University Hospital, Amiens, France; 9 Internal Medicine, Lille University Hospital, Lille, France; 10 Internal Medicine, Avicenne Hospital, Assistance Publique des Hôpitaux de Paris, Bobigny, France; 11 Internal Medicine, Cochin Hospital, Assistance Publique des Hôpitaux de Paris, Paris, France; 12 Rheumatology, Montpellier University Hospital, Montpellier, France; 13 Rheumatology, Rennes University Hospital, Rennes, France; 14 Rheumatology, Orléans Hospital, Orléans, France; 15 Internal Medicine, Lariboisière Hospital, Assistance Publique des Hôpitaux de Paris, Paris, France; 16 Rheumatology, Rouen University Hospital, Rouen, France; 17 Center of Clinical Epidemiology, Hotel Dieu Hospital, Assistance Publique des Hôpitaux de Paris, Institut National de la Santé et de la Recherche Médicale U378, University of Paris Descartes, Faculty of Medicine, Paris, France; Centro di Riferimento Oncologico, IRCCS National Cancer Institute, Italy

## Abstract

**Objectives:**

To analyze the clinical and immunological characteristics at enrollment in a large prospective cohort of patients with primary Sjögren's syndrome (pSS) and to investigate the association between serum BAFF, beta2-microglobulin and free light chains of immunoglobulins and systemic disease activity at enrollment.

**Methods:**

Three hundred and ninety five patients with pSS according to American-European Consensus Criteria were included from fifteen centers of Rheumatology and Internal Medicine in the “Assessment of Systemic Signs and Evolution of Sjögren's Syndrome” (ASSESS) 5-year prospective cohort. At enrollment, serum markers were assessed as well as activity of the disease measured with the EULAR Sjögren's Syndrome Disease Activity Index (ESSDAI).

**Results:**

Patient median age was 58 (25^th^–75^th^: 51–67) and median disease duration was 5 (2–9) years. Median ESSDAI at enrollment was 2 (0–7) with 30.9% of patients having features of systemic involvement. Patients with elevated BAFF, beta2-microglobulin and kappa, lambda FLCS had higher ESSDAI scores at enrollment (4 [2]–[11] vs 2 [0–7], *P* = 0.03; 4 [1]–[11] vs 2 [0–7], *P*< 0.0001); 4 [2]–[10] vs 2 [0–6.6], *P*< 0.0001 and 4 [2–8.2] vs 2 [0–7.0], *P* = 0.02, respectively). In multivariate analysis, increased beta2-microglobulin, kappa and lambda FLCs were associated with a higher ESSDAI score. Median BAFF and beta2-microglobulin were higher in the 16 patients with history of lymphoma (1173.3(873.1–3665.5) vs 898.9 (715.9–1187.2) pg/ml, *P* = 0.01 and 2.6 (2.2–2.9) vs 2.1 (1.8–2.6) mg/l, *P* = 0.04, respectively).

**Conclusion:**

In pSS, higher levels of beta2-microglobulin and free light chains of immunoglobulins are associated with increased systemic disease activity.

## Introduction

In autoimmune diseases, using biomarkers to allow the identification of patients at risk of flares or complications remains an unmet need. These patients would thus benefit from a tightened follow-up and earlier use of immunosuppressant agents to limit end-organ damage and mortality.

Patients with primary Sjögren's syndrome (pSS), the most common systemic autoimmune disease after rheumatoid arthritis, would particularly benefit from the identification of disease activity and prognostic markers. The hallmarks of pSS involve a disabling dry syndrome associated with asthenia and pain, but the course of the disease cannot be predicted and one third of the patients will develop systemic manifestations [1], [2]. Moreover, 5% to 10% of patients will develop lymphoma [3]–[5], and only few predictive factors of this severe complication have been identified to date. Lastly, conversely to many other autoimmune diseases, the use of acute phase reactants is limited because elevation of C-reactive protein is rare and the elevation of the erythrocyte sedimentation rate is frequently due to hypergammaglobulinemia.

A very important milestone to allow the identification of biomarkers was accomplished by the establishment of an international consensus disease activity score, named the ESSDAI (EULAR Sjögren's Syndrome Disease Activity Index) [6]. This quantitative score includes 12 domains corresponding to various clinical and biological features. The nature of each of these involvements was consensually defined. The pathogenesis of pSS involves both genetic and environmental factors, which trigger innate and adaptive immunity, including T and B lymphocytes (2). Concordant data from several groups have emphasized the role of type-I interferon-inducible proteins, including BAFF (B-cell Activation of the TNF Family, also named BLyS) in the activation of B lymphocytes [7]. Elevated BAFF has been evidenced in the serum and target organs of pSS (salivary glands, conjunctival epithelial cells of the ocular surface). BAFF is secreted not only by monocytes and dendritic cells but also by T and B lymphocytes and salivary gland epithelial cells in pSS [8]–[11]. B lymphocytes in pSS have a decreased BAFF-R expression, possibly related to BAFF overexpression and this is more marked in patients with systemic features [12]. Lymphoma is thought to result from persistent activation of B-cells, to which the increased BAFF concentration might contribute [13]. Previous studies have suggested that the activation of B-cells could contribute to systemic complications in pSS [14]–[19] but they were retrospective or cross-sectional, concerned one isolated serum marker (beta2-microglobulin [18] or BAFF [19] for example), past or current systemic complications, without concomitant assessment of disease activity and serum markers. Last, until the recent establishment of the ESSDAI, disease activity was almost exclusively defined by the presence or absence of systemic manifestations.

We have therefore set up a multicenter 5-year prospective cohort and associated biobank named ASSESS (Assessment of Systemic Signs and Evolution in Sjögren's Syndrome) in France in order to determine whether serum BAFF, beta2-microglobulin, free light chains of immunoglobulins (primary objective) or other serum or mRNA markers (secondary objectives) could predict the occurrence of systemic complications including lymphoma.

Since the patients have not achieved their expected 5 years of follow-up, the objectives of the present study are to describe the baseline characteristics of the ASSESS cohort and to investigate the association of serum BAFF, beta2-microglobulin, kappa and lambda free light chains of immunoglobulins (FLCs) with systemic disease activity scored by the ESSDAI, concomitantly evaluated at inclusion in the prospective ASSESS cohort.

## Patients and Methods

### Patients

The ASSESS national multi-center prospective cohort (Assessment of Systemic Signs and Evolution in Sjögren's Syndrome) was set up in 2006 with a grant of the French Ministry of Health (Programme Hospitalier de Recherche Clinique 2005 P060228). Its primary objective was to identify valuable predictive factors of systemic complications and lymphoma in pSS during a 5-year prospective follow-up.

The study, promoted by the Assistance Publique des Hôpitaux de Paris, was approved by the Ethics Committee of Hôpital Bichat and the “Commission Nationale Informatique et Libertés” in 2006. All patients gave their informed written consent.

Four hundred and ten patients with pSS fulfilling American-European Consensus Criteria (AECG) were consecutively included from fifteen tertiary centers for autoimmune diseases between 2006 and 2009.

The present and previous systemic complications including skin, articular, lung, kidney, peripheral and central nervous system, muscular involvement and vasculitis, as well as lymphoma, were recorded. The diagnosis and the histological subtypes of previous lymphomas were confirmed by reanalysis of the medical and histological records by one of us (XM).

A thoroughly standardized paper clinical report form (CRF) was prospectively completed by participating clinicians each year for a total of 5 years. Disease systemic activity was assessed by the ESSDAI. This quantitative score (range: 0 to 123) has 12 domains [6]. For instance, pSS-related articular involvement as defined by the articular domain of the ESSDAI corresponds to arthralgias in hands, wrists, ankles and feet accompanied by morning stiffness (>30 min) and/or synovitis. The biological domain corresponds to the presence of hypocomplementemia, clonal component, cryoglobulinemia, hypergammaglobulinemia or high IgG level, recent onset hypogammaglobulinemia or recent decrease of IgG level (<5 g/L). The haematological domain corresponds to the presence of a cytopenia of auto-immune origin (neutropenia, anemia, thrombocytopenia, or lymphopenia [6].

The ESSPRI score (EULAR Sjögren's Syndrome Patient Related Index), a patient reported outcome multinational consensus score evaluating dryness, fatigue, and pain, was completed by all patients each year [20]. Dryness was objectively assessed at enrollment and after 2 and 5 years of follow-up using unstimulated salivary flow (USF) and Schirmer's test. After monitoring, data were included in an electronic database.

### Assessment of serum markers

DNA, blood RNA and serum samples were obtained at enrollment. All biological samples were immediately frozen, stored and shipped to the Centre de Ressources Biologiques of Bichat Hospital, which has obtained the French Association for Quality Insurance (AFAQ) (certification number 2009/34457) according to the norm 96900. Blood RNA was collected using a Paxgene tube, extracted using a Qiagen extraction kit, and stored at −80°C. The NanoDrop ND-1000 UV-Vis spectrophotometer was used to determine the quantity and the purity of DNA that have been isolated. Agilent's 2100 Bioanalyzer was used to determine both RNA concentration and integrity showing two distinct ribosomal peaks corresponding to either to 18S and 28S. The 28S/18S calculated ratio was used to serve as an indication of RNA integrity. Serum markers were assessed centrally and blindly from any clinical or other biological data. Rheumatoid factor (RF) was assessed using Enzyme-Linked Immunosorbent Assay (ELISA), C3 and C4 using nephelometry (decreased C3 and C4 defined as a value below 0.8 and 0.15 g/l, respectively). Beta2-microglobulin, total Ig levels, kappa and lambda FLCs of Igs were assessed by nephelometry using the Freelite kit (Binding Site), BAFF was measured using ELISA (Quantikine kit, R&D Systems). Increased concentrations of beta2-microglobulin, kappa and lambda free light chains, or abnormal kappa to lambda ratio were defined according to the manufacturer (greater than 2.3 mg/l, 19.4 mg/l, 26.3 mg/l, and less than 0.26 or over 1.65, respectively). Serum elevated BAFF was defined by a value greater or equal to the mean (916.9±271.2 pg/ml )+2 standard deviations (1459.3 pg/ml) of 80 age and sex-matched control subjects (subjects with dry symptoms but without any autoantibody and without lymphocytic infiltrates on minor salivary gland biopsy). CD4 and CD8 T cell counts were determined using flow cytometry. CD4+ T lymphocytopenia was defined by an absolute CD4 count lower than 300 cells/µl [21].

### Statistical analysis

Continuous data are presented as medians with interquartile ranges. We used nonparametric tests to analyze continuous variables, despite the large number of patients, because the distribution of data was uneven. The Mann-Whitney U test was used to compare continuous data and the chi-squared test to compare nominal data. Correlations were studied with Spearman's rank test. Biological markers associated with the ESSDAI by univariate analysis were included in multivariate linear and logistic regression models. All statistical analyses were performed with Stata SE 9.2 (Stata Corporation, College Station, TX, USA).

## Results

### Characteristics of patients and serum levels of BAFF, beta2-microgobulin and immunoglobulin free light chains

410 patients were included in the cohort. Fifteen patients were excluded from the cohort since they did not fulfill the AECG criteria for pSS (n = 14) or withdrew consent (n = 1). The characteristics of the 395 patients with pSS are summarized in Table 1. 93.6% of patients were women, median age was 58 (51–67) years and median disease duration was 5 (2–9) years. 59.2% of patients were anti-SSA positive and 33.5% anti-SSB positive. 41.1% of patients were rheumatoid factor-positive. 34.1% of the patients had a history of systemic involvement. 30.9% of patients had active systemic involvement at enrollment. The median ESSDAI score was 2 (0–7.0). At enrollment, 23.7% were treated with corticosteroids (median dosage 5 mg/day), 30.7% with hydroxychloroquine (HQ), 9.4% with an immunosuppressant other than HQ or corticosteroids (Table 1). Median levels of BAFF, beta2- microglobulin and free light chains of immunoglobulin levels are reported in Table 1.

**Table 1:** Characteristics of patients at enrollment in the ASSESS cohort.

Median age (years)	58 (51–67)
Male/female	93.6/6.4%
Disease duration (years)	5 (2–9)
Decreased salivary flow at enrollment	162/327 (47.5%)
Focus score ≥ 1	318/352 (87.8%)
Previous systemic involvement	34.1%
Previous lymphoma	4.7%
Active current systemic involvement	30.9%
No active nor previous involvement	28.8%
Enlarged parotids at enrollment	11.4%
Purpura at enrollment	2.3%
ESSDAI	2.0 (0.–7.0)
ESSPRI	5.7 (4.0–7.0)
Treatment with corticosteroids at enrollment	23.7%
Median dosage	5 mg/day
Treatment with hydroxychloroquine at enrollment	30.7%
Treatment with other immunosuppressants at enrollment	
MTX	5.1%
LEF	0.5%
AZA	1.5%
MMF	1.3%
RTX in the 6 months preceding enrollment	1%
Anti-SSA positive antibodies	59.2%
Anti-SSB positive antibodies	33.5%
RF-positive	153/372 (41.1%)
Cryoglobulinemia	57/335 (17.0%)
Low C4 (< 0.15 g/l)	72/371 (19.4%)
IgG, g/l; high IgG (>16 g/l)	12.5 (9.9–16.8); 29.3%
Serum monoclonal immunoglobulin	13.1%
CD4+ T cell lymphocytopenia (<300 cells/µl )	9.8%
CD4/CD8 < 0.8	19/338 (5.6%)
BAFF (pg/ml)	909.0 (719.1–1196.1)
Beta2-microglobulin (mg/l)	2.2 (1.8–2.6)
Elevated levels, %	38.4%
Immunoglobulin kappa free light chains (mg/l)	13.5 (10.1–19.2)
Elevated levels, %	24.4%
Lambda free light chains (mg/l)	13.2 (9.8–18.8)
Elevated levels, %	10.1
Abnormal kappa to lambda ratio, %	7.4

Results are expressed as medians (25^th^–75^th^ percentiles) unless otherwise specified. MTX: methotrexate; LEF: leflunomide; AZA: azathioprine; MMF: mycophenolate mofetil; RTX: rituximab; RF: rheumatoid factor.

For all the further analyses on markers of B-cell activation, two patients were excluded since they had been prescribed rituximab within 12 months before enrollment in the cohort, a treatment which might especially affect serum BAFF levels [22], [23]. Patients with anti-SSA or with both anti-SSA and anti-SSB autoantibodies had significantly higher levels of BAFF, beta2-microgobulin and immunoglobulin free light chains (data not shown). Serum BAFF level was weakly but significantly correlated with serum beta2-microglobulin (r = 0.2, *P*<0.0001), but not with IgG, kappa and lambda immunoglobulin free light chains or RF concentrations (data not shown).

### Association between the systemic disease activity at enrollment in the ASSESS cohort and serum levels of BAFF, beta2-microgobulin and immunoglobulin free light chains

The median ESSDAI was 2 (0–7.0). The proportion of patients who, at enrollment, had active disease, defined by the presence of at least low, or moderate or high disease activity in the various domains of the ESSDAI [6], is indicated in Table 2. Patients with anti-SSA/SSB, or increased markers of BAFF, beta2-microglobulin, immunoglobulin kappa and lambda free lights chains had a significantly higher ESSDAI. Multivariate linear regression analysis indicated that only beta2-microglobulin and kappa FLC concentrations were associated with the ESSDAI (Table 3).

**Table 2:** Patients with active disease in the various ESSDAI domains at enrollment.

Domain	Proportion of patients with disease activity
Skin	4.2%
Pulmonary	14.4%
Renal	2.8%
Articular	18.6%
Muscular	3.3%
Peripheral neuropathy	9.6%
Central nervous system	2.0%
Glandular	12.1%
Constitutional	4.1%
Haematological	15.6%
Lymphadenopathy	2.4%
Biological	37.4%

Clinical involvement corresponding to each of these twelve domains is defined according to the ESSDAI (ref 6).

**Table 3:** Association between autoantibodies, serum markers and ESSDAI.

	ESSDAI	*P* value, univariate	*P* value, multivariate
Anti-SSA +	3 (1–8)	0.02	0.6
Anti-SSA −	2 (0–6)		
RF +	3 (1–8)	0.2	0.2
RF −	2 (0–7)		
Elevated BAFF	4 (2–11)	0.03	0.1
Normal BAFF	2 (0–7)		
Elevated β2	4 (1–11)	<10^−4^	0.03
Normal β2	2 (0–7)		
Elevated κ	4 (2–10)	<10^−4^	0.02
Normal κ	2 (0–6.6)		
Elevated λ	4 (2–8.2)	0.02	0.5
Normal λ	2 (0–7.0)		

αSSA +: anti-SSA-positive(anti-SSA alone or anti-SSA plus anti-SSB); RF: rheumatoid factor; β2: beta2-microglobulin; κ: kappa free light chains of immunoglobulins; λ: lambda free light chains of immunoglobulins.

Results are expressed as medians (25^th^–75^th^).

Conversely, the ESSPRI, which evaluates patient's dryness, fatigue, and pain, was not associated with increases in BAFF, beta2-microglobulin, kappa and lambda FLCs. These serum markers were not increased either in patients with decreased unstimulated salivary flow or abnormal Schirmer's test (data not shown).

### Increased serum BAFF and beta2-microglobulin in patients with a history of lymphoma

The cohort included 16 patients with a history of lymphoma, without any rituximab treatment in the past 12 months, 10 of whom had been treated with rituximab more than 1 year ago. The median time between the diagnosis of lymphoma and enrollment in the cohort was 6 years (0–11). Baseline serum BAFF and beta2- microglobulin were significantly higher in these 16 patients with history of lymphoma than in the others (1173.3 (873.1–3665.5) vs 898.9 pg/ml (715.9–1187.2), *P* = 0.01 and 2.6 (2.2–2.9) vs 2.1 (1.8–2.6) mg/, *P* = 0.04, respectively). A history of lymphoma was not associated with serum FLCs, an abnormal kappa to lambda ratio or the RF concentration (data not shown). After exclusion of the 3 patients not in remission of lymphoma, the disease activity at enrollment (ESSDAI: 4.0 (1.2–8.6)) of the 13 patients with a history of lymphoma and in remission was not significantly higher than that of the other patients (2.0 (0–6.8), *P* = 0.5). Regarding the 3 main biological factors associated with a risk of lymphoma [24]–[26], a low C4 and a CD4/CD8 ratio lower than 0.8, were significantly more frequent in patients with a history of lymphoma and there was a trend towards an association with cryoglobulinemia (Table 4).

**Table 4:** Characteristics associated with a history of lymphoma.

	Patient with history of lymphoma (n = 16)	Patients without history of lymphoma (n = 377)	*P* value
BAFF, pg/ml	1173.3(873.1–3665.5)	898.9 (715.9–1187.2)	0.01
Beta2- microglobulin, mg/l	2.6 (2.2–2.9)	2.1 (1.8–2.6)	0.04
Cryoglobulinemia	35.7%	16.7%	0.2
Low C4 (< 0.15 g/l)	57.1%	17.9%	0.003
CD4+T lymphocytopenia (<300 cells/µl)	28.5%	8.7%	0.06
CD4/CD8 ratio<0.8	28.5%	4.8%	0.01

Results are expressed as medians (25^th^–75^th^ percentiles) unless otherwise specified.

## Discussion

The present study reports the clinical and immunological characteristics of patients with pSS at enrollment in a large prospective cohort and demonstrates the association between systemic disease activity assessed by the ESSDAI and beta2-microglobulin and free light chains of immunoglobulins.

First, this study reports the characteristics of patients at enrollment in a large multicenter 5-year prospective cohort. A better knowledge of the clinical outcome, the course of disease activity and the risk factors of lymphoma in pSS require the setting up of cohorts with biobanks [27]. Three large prospective cohorts, including the present one, have already started to enroll patients with pSS. The clinical manifestations and immunological patterns in the ASSESS cohort are representative of the patients with pSS referred to rheumatology or internal medicine centers. In addition, only a limited proportion of the patients were treated with low doses of corticosteroids, or immunosuppressants other than hydroxychloroquine. Thus, the analysis of serum markers was not flawed by the use of immunosuppressants. The storage of all samples in a biological resource center and the central processing and analysis, blinded from the clinical data, ensures the quality of the stored sera and resulting data. Last, this is the first large cohort in which ESSDAI scores of clinical activity and different serum biomarkers have been evaluated simultaneously.

However, this study has some limitations. It is currently a cross-sectional study and it is necessary to wait for years before being able to evaluate the interest of these biomarkers as predictive factors of complications. In addition, among the 12 domains of the ESSDAI score, there is one biological domain which takes into account different parameters than those assessed in this study: levels of gammaglobulins, IgG and complement, and presence of monoclonal component and cryoglobulinemia. However, removing this domain from the ESSDAI represents a methodological nonsense since the ESSDAI has been built with all these 12 domains with a specific weight calculated for each of them. Thus, it was not possible to compare the value as disease activity markers of BAFF, beta2-microglobulin and free light chains of immunoglobulins with that of the biological domain (gammaglobulins, Ig or complement).

There is clearly a rationale to investigate serum BAFF, beta2-microglobulin and free light chains of immunoglobulins as disease activity markers in pSS. Retrospective studies have shown that beta2-microglobulin and free light chains levels are associated with extra-glandular complications [14]–[18]. Recently, 2 retrospective studies including 78 and 76 pSS patients showed in univariate analysis a significant correlation between the ESSDAI and serum beta2-microglobulin and BAFF, respectively [18], [19]. This study extends these findings by analyzing in a multivariate manner, in a large cohort, the relative contribution of both markers with regards to anti-SSA/SSB, RF, and free light chains of immunoglobulins. Patients with increases in BAFF, beta2-microglobulin or free light chains of immunoglobulins, had an approximately two-fold increase in their ESSDAI value. No such an association was observed with autoantibodies, since RF was not associated in univariate analysis and anti-SSA/SSB antibodies were not associated in multivariate analysis with the ESSDAI. In addition, positivity for anti-SSA/SSB is genetically determined [28] and a previous study showed that their quantitative levels were not associated with extraglandular involvement [29]. Of note, there was no association between the studied serum markers and unstimulated salivary flow or Schirmer's test or with the new international consensus patient-related outcome score, the ESSPRI, which evaluates patient's pain, fatigue and symptoms of dryness.

The primary objective of this prospective cohort is to identify factors predicting the occurrence of systemic complications and especially of lymphoma. It was recently demonstrated in a retrospective study that patients with pSS and lymphoma had higher levels of BAFF at the time of diagnosis of lymphoma [19]. Interestingly, patients with a history of lymphoma included in ASSESS cohort had elevated serum concentrations of BAFF and beta2-microglobulin. Elevated BAFF and beta2-microglobulin concentrations in patients with history of lymphoma were not due to greater systemic disease activity at enrollment. Elevated serum BAFF and beta2-microglobulin are associated with a poor prognosis in non-Hodgkin lymphomas that occur independently from autoimmunity [13]. But 13 out of our 16 patients with a history of lymphoma had been in remission for 6 years before enrollment and still had elevated serum BAFF and beta2-microglobulin concentrations at inclusion in the cohort. This might suggest a genetic origin of such a persistent increase in BAFF level. The –871 C→T single nucleotide gene polymorphism (SNP) has not been associated with pSS but with the serum BAFF concentration in patients with pSS [30], [31], with mixed cryoglobulinemia [32], with familial chronic lymphoproliferations [33], [34], and in patients with idiopathic thrombopenic purpura [35]. However, the study of a haplotype block including four SNPs (−2841 T→C, −2704 T→C, −2701 T→A, −871 C→T), suggested that the −871 T allele was unlikely to be a sole, major determinant of serum BAFF levels [31]. It is now necessary to assess the relevance of serum BAFF and beta2-microglobulin concentrations as markers predicting new cases of lymphoma during the 5-year prospective follow-up of the ASSESS cohort.

## Conclusion

The association of serum beta2-microglobulin and free light chains of immunoglobulins with systemic disease activity offers clinical perspectives to personalize healthcare in pSS. Should these biomarkers be validated in the prospective ASSESS cohort, they could help to identify pSS patients at risk of systemic complications who need closer clinical monitoring and facilitate the selection of patients who may benefit from more aggressive immunosuppressive therapy to avoid these complications.

## References

[1] Fox RI (2005) Sjögren's syndrome. Lancet. 23–29 366: 321–31. 10.1016/S0140-6736(05)66990-5 16039337

[2] Mariette X, Gottenberg JE (2010) Pathogenesis of Sjögren's syndrome and therapeutic consequences. Curr Opin Rheumatol 22: 471–7. 20671520 10.1097/BOR.0b013e32833c36c5

[3] Theander E, Henriksson G, Ljungberg O, Mandl T, Manthorpe R, et al (2006) Lymphoma and other malignancies in primary Sjögren's syndrome: a cohort study on cancer incidence and lymphoma predictors. Ann Rheum Dis. 65: 796–803. 10.1136/ard.2005.041186 PMC1798187 16284097

[4] Ioannidis JP, Vassiliou VA, Moutsopoulos HM (2002) Long-term risk of mortality and lymphoproliferative disease and predictive classification of primary Sjögren's syndrome. Arthritis Rheum. 46: 741–7. 10.1002/art.10221 11920410

[5] Kassan SS, Thomas TL, Moutsopoulos HM, Hoover R, Kimberly RP, et al (1978) Increased risk of lymphoma in siccasyndrome.Ann Intern Med. 89: 888–92. 10.7326/0003-4819-89-6-888 102228

[6] Seror R, Ravaud P, Bowman SJ, Baron G, Tzioufas A, et al (2011) EULAR Sjogren's syndrome disease activity index: development of a consensus systemic disease activity index for primary Sjogren's syndrome. Ann Rheum Dis. 2010; 69: 1103–9. Erratum in: Ann Rheum Dis. 70: 880. 10.1136/ard.2009.110619 PMC2937022 19561361

[7] Mackay F, Groom JR, Tangye SG (2007) An important role for B-cell activation factor and B cells in the pathogenesis of Sjögren's syndrome. Curr Opin Rheumatol. 19: 406–13. 10.1097/BOR.0b013e328277ef4c 17762603

[8] Jonsson MV, Szodoray P, Jellestad S, Jonsson R, Skarstein K (2005) Association between circulating levels of the novel TNF family members APRIL and BAFF and lymphoid organization in primary Sjögren's syndrome. J Clin Immunol. 25: 189–201. 10.1007/s10875-005-4091-5 15981083

[9] Mariette X, Roux S, Zhang J, Bengoufa D, Lavie F, et al (2003) The level of BLyS (BAFF) correlates with the titre of autoantibodies in human Sjögren's syndrome.Ann Rheum Dis. 62: 168–71. 10.1136/ard.62.2.168 PMC1754442 12525388

[10] Lavie F, Miceli-Richard C, Ittah M, Sellam J, Gottenberg JE, et al (2008) B-cell activating factor of the tumour necrosis factor family expression in blood monocytes and T cells from patients with primary Sjögren's syndrome. Scand J Immunol. 67: 185–92. 10.1111/j.1365-3083.2007.02049.x 18201372

[11] Daridon C, Devauchelle V, Hutin P, Le Berre R, Martins-Carvalho C, et al (2007) BAFF-modulated repopulation of B lymphocytes in the blood and salivary glands of rituximab-treated patients with Sjögren's syndrome. Arthritis Rheum. 56: 1464–77. 10.1002/art.22603 17469105

[12] Sellam J, Miceli-Richard C, Gottenberg JE, Ittah M, Lavie F, et al (2007) Decreased B cell activating factor receptor expression on peripheral lymphocytes associated with increased disease activity in primary Sjögren's syndrome and systemic lupus erythematosus. Ann Rheum Dis. 66: 790–7. 10.1136/ard.2006.065656 PMC1954659 17185325

[13] Novak AJ, Grote DM, Stenson M, Ziesmer SC, Witzig TE, et al (2004) Expression of BLyS and its receptors in B-cell non-Hodgkin lymphoma: correlation with disease activity and patient outcome. Blood.15 104: 2247–53. 10.1182/blood-2004-02-0762 15251985

[14] Gottenberg JE, Busson M, Cohen-Solal J, Lavie F, Abbed K, et al (2005) Correlation of serum B lymphocyte stimulator and beta2 microglobulin with autoantibody secretion and systemic involvement in primary Sjogren's syndrome. Ann Rheum Dis. 64: 1050. 10.1136/ard.2004.030643 PMC1755548 15640273

[15] Gottenberg JE, Aucouturier F, Goetz J, Sordet C, Jahn I, et al (2007) Serum immunoglobulin free light chainassessment in rheumatoid arthritis and primary Sjogren'ssyndrome. Ann Rheum Dis. 66: 23–7. 10.1136/ard.2006.052159 PMC1798389 16569685

[16] Michalski JP, Daniels TE, Talal N, Grey HM (1975) Beta2 microglobulin and lymphocytic infiltration in Sjögren's syndrome. N Engl J Med. 293: 1228–31. 10.1056/NEJM197512112932404 52841

[17] Moutsopoulos HM, Steinberg AD, Fauci AS, Lane HC, Papadopoulos NM (1983) High incidence of free monoclonal lambda light chains in the sera of patients with Sjogren's syndrome. J Immunol 130: 2663–5. 6406594

[18] Pertovaara M, Korpela M (2011) Serum beta2-microglobulin correlates with the new ESSDAI in patients with Sjögren's syndrome. Ann Rheum Dis 70: 2236–2237. 21628309 10.1136/ard.2011.153098

[19] Quartuccio L, Salvin S, Fabris M, Maset M, Pontarini E, et al.. (2012) BLyS upregulation in Sjögren's syndrome associated with lymphoproliferative disorders, higher ESSDAI score and B-cell clonal expansion in the salivary glands. Rheumatology August epub ahead of print. 10.1093/rheumatology/kes180 22879463

[20] Seror R, Ravaud P, Mariette X, Bootsma H, Theander E, et al (2011) EULAR Sjogren's Syndrome Patient Reported Index (ESSPRI): developmentof a consensus patient index for primary Sjogren'ssyndrome. Ann Rheum Dis 70: 968–72. 10.1136/ard.2010.143743 21345815

[21] Smith DK, Neal JJ, Holmberg SD (1993) Unexplained opportunistic infections and CD4+ T-lymphocytopenia without HIV infection: an investigation of cases in the United States. N Engl J Med. 328: 373–379. 10.1056/NEJM199302113280601 8093633

[22] Cambridge G, Stohl W, Leandro MJ, Migone TS, Hilbert DM, et al (2006) Circulating levels of B lymphocyte stimulator in patients with rheumatoid arthritis following rituximab treatment: relationships with B cell depletion, circulating antibodies, and clinical relapse. Arthritis Rheum. 54: 723–32. 10.1002/art.21650 16508933

[23] Lavie F, Miceli-Richard C, Ittah M, Sellam J, Gottenberg JE, et al (2007) Increase of B cell-activating factor of the TNF family (BAFF) after rituximab treatment: insights into a new regulating system of BAFF production. Ann Rheum Dis. 66: 700–3. 10.1136/ard.2006.060772 PMC1954605 17040963

[24] Voulgarelis M, Moutsopoulos HM (2008) Mucosa-associated lymphoid tissue lymphoma in Sjögren's syndrome: risks, management, and prognosis. Rheum Dis Clin North Am. 34: 921–33. 10.1016/j.rdc.2008.08.006 18984412

[25] Tzioufas AG, Boumba DS, Skopouli FN, Moutsopoulos HM (1996) Mixed monoclonal cryoglobulinemia and monoclonal rheumatoid factor cross-reactive idiotypes as predictive factors for the development of lymphoma in primary Sjögren's syndrome. Arthritis Rheum. 39: 767–72. 10.1002/art.1780390508 8639173

[26] Theander E, Henriksson G, Ljungberg O, Mandl T, Manthorpe R, et al (2006) Lymphoma and other malignancies in primary Sjögren's syndrome: a cohort study on cancer incidence and lymphoma predictors. Ann Rheum Dis. 65: 796–803. 10.1136/ard.2005.041186 PMC1798187 16284097

[27] Gottenberg JE, Mariette X (2012) Primary Sjogren's Syndrome: Time for Prospective Cohorts. Curr Pharm Biotechnol. 1 13(10): 2022–5. 10.2174/138920112802273128 22208655

[28] Gottenberg JE, Busson M, Loiseau P, Cohen-Solal J, Lepage V, et al (2003) In primary Sjögren's syndrome, HLA class II is associated exclusively with autoantibody production and spreading of the autoimmune response.Arthritis Rheum. 48: 2240–5. 10.1002/art.11103 12905478

[29] Candon S, Gottenberg JE, Bengoufa D, Chatenoud L, Mariette X (2009) Quantitative assessment of antibodies to ribonucleoproteins in primary Sjögren syndrome: correlation with B-cell biomarkers and disease activity. Ann Rheum Dis. 68: 1208–12. 10.1136/ard.2008.095257 18713786

[30] Gottenberg JE, Sellam J, Ittah M, Lavie F, Proust A, et al (2006) No evidence for an association between the −871 T/C promoter polymorphism in the B-cell-activating factor gene and primary Sjögren's syndrome. Arthritis Res Ther. 8: R30. 10.1186/ar1884 PMC1526574 16507129

[31] Nossent JC, Lester S, Zahra D, Mackay CR, Rischmueller M (2008) Polymorphism in the 5′ regulatory region of the B-lymphocyte activating factor gene is associated with the Ro/La autoantibody response and serum BAFF levels in pirmary Sjögren's syndrome. Rheumatology 47: 1311–1316. 18617551 10.1093/rheumatology/ken246

[32] Gragnani L, Piluso A, Giannini C, Caini P, Fognani E, et al (2011) Genetic determinants in hepatitis C virus-associated mixed cryoglobulinemia: role of polymorphic variants of BAFF promoter and Fcgamma receptors. Arthritis Rheum 63: 1446–51. 21538321 10.1002/art.30274

[33] Novak AJ, Slager SL, Fredericksen ZS, Wang AH, Manske MM, et al (2009) Genetic variation in B-cell-activating factor is associated with an increased risk of developing B-cell non-Hodgkin lymphoma. Cancer Res. 15 69: 4217–24. 10.1158/0008-5472.CAN-08-4915 PMC2743448 19383901

[34] Novak AJ, Grote DM, Ziesmer SC, Kline MP, Manske MK, et al (2006) Elevated serum B-lymphocyte stimulator levels in patients with familial lymphoproliferative disorders. J Clin Oncol. 20 24: 983–7. 10.1200/JCO.2005.02.7938 16432079

[35] Emmerich F, Bal G, Barakat A, Milz J, Mühle C, et al (2007) High-level serum B-cell activating factor and promoter polymorphisms in patients with idiopathic thrombocytopenic purpura. Br J Haematol 136: 309–1. 10.1111/j.1365-2141.2006.06431.x 17156395

